# Stimuli-Responsive Graphene Nanohybrids for Biomedical Applications

**DOI:** 10.1155/2019/9831853

**Published:** 2019-04-02

**Authors:** Dinesh K. Patel, Yu-Ri Seo, Ki-Taek Lim

**Affiliations:** ^1^The Institute of Forest Science, Kangwon National University, Chuncheon 24341, Republic of Korea; ^2^Department of Biosystems Engineering, College of Agriculture and Life Sciences, Kangwon National University, Chuncheon 24341, Republic of Korea

## Abstract

Stimuli-responsive materials, also known as smart materials, can change their structure and, consequently, original behavior in response to external or internal stimuli. This is due to the change in the interactions between the various functional groups. Graphene, which is a single layer of carbon atoms with a hexagonal morphology and has excellent physiochemical properties with a high surface area, is frequently used in materials science for various applications. Numerous surface functionalizations are possible for the graphene structure with different functional groups, which can be used to alter the properties of native materials. Graphene-based hybrids exhibit significant improvements in their native properties. Since functionalized graphene contains several reactive groups, the behavior of such hybrid materials can be easily tuned by changing the external conditions, which is very useful in biomedical applications. Enhanced cell proliferation and differentiation of stem cells was reported on the surfaces of graphene-based hybrids with negligible cytotoxicity. In addition, pH or light-induced drug delivery with a controlled release rate was observed for such nanohybrids. Besides, notable improvements in antimicrobial activity were observed for nanohybrids, which demonstrated their potential for biomedical applications. This review describes the physiochemical properties of graphene and graphene-based hybrid materials for stimuli-responsive drug delivery, tissue engineering, and antimicrobial applications.

## 1. Introduction

**Figure 1:**
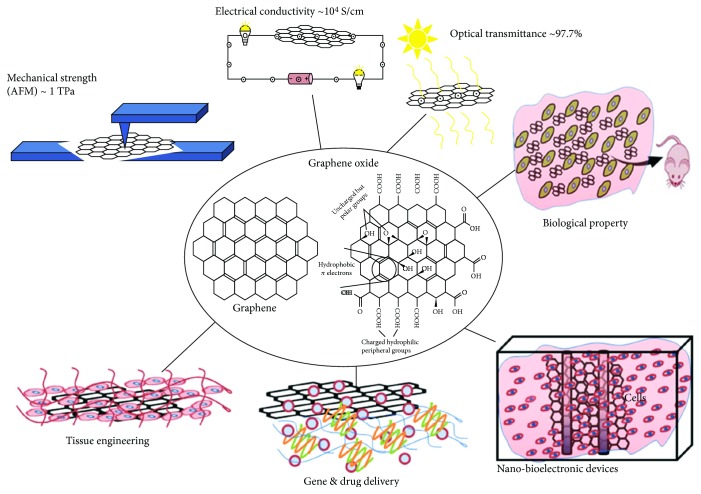
Schematic overview of various applications of graphene. Graphene-based nanomaterials have been explored for various nonmedical and biomedical applications due to their excellent mechanical, electrical, and optical properties [41].

Nowadays, on-demand release of active materials in desired areas has drawn tremendous attention in the rapidly developing field of materials science. For this purpose, stimuli-responsive materials (SRMs), which are also known as smart materials, are frequently used. They can change their shapes or dimensions in the presence of external stimuli such as electric field [1, 2], magnetic field [3, 4], temperature [5–7], pH [8], light [9–12], pressure [13], solvent [14], and moisture [15]. Stimuli-responsive polymers can be used in electrochemical devices [16], biomimetic devices [17], actuators and sensors [18], active sound-absorbing materials, smart textiles and apparel [19], intelligent medical instruments and auxiliaries [20, 21], and flexible devices [19]. Multiple cooperative interactions such as loss of hydrogen bonding and progressive ionization in polymer units are the key factors for such effects when the smart materials are exposed to external stimuli. Several polymers such as poly(ethylene oxide) (PEO), poly(propylene oxide) (PPO), poly(*N*-vinylcaprolactam), poly(*N*-isopropylacrylamide), poly(*N*,*N*^″^*-*diethylacrylamide), and other copolymers are frequently used as a smart material for various biomedical applications [22]. For biomedical applications, materials should be biocompatible and biodegradable and should not show any immune response in biological conditions. In addition, the materials should have adequate mechanical strength to support the proper growth of cells [23]. The properties of smart materials can be easily tuned by changing their structures or incorporating suitable fillers in their matrices. Metal and their oxides, clay with different modifications, nanocellulose, zeolites, and carbon in different forms such as fullerenes, carbon nanotubes, and graphite are frequently used to enhance the properties of native polymers [24–29]. According to the requirements, these fillers can be used to alter the properties of the matrix materials. Among these, graphite has drawn significant attention in the field of materials science as a reinforcing agent owing to its unique structural, thermal, mechanical, electrical, and biological properties [30–32]. Graphene is a single layer of sp^2^-bonded carbon atoms with a honeycomb morphology. The higher surface area of graphene facilitates effective binding with several drugs through various interactions and is frequently used in targeted and controlled drug delivery applications [33, 34]. The hydrophobic nature of graphene restricts its use in polar environments. This problem can be overcome by rendering graphene hydrophilic by inserting different polar groups such as hydroxyl, epoxy, and carboxyl groups through surface functionalization. The presence of different functional groups in graphene oxide (GO) provides a platform for surface functionalization that can be used for various applications. Moreover, structural defects are created by oxidation, which lead to a decrease in their electrical property [35]. The structural defect is very useful in energy band gap applications. However, the electrical property can be restored by the reduction of GO, which is carried out through heating at higher temperatures in inert conditions or using various reducing agents such as hydrazine and alkaline media [36]. Extraordinary physiochemical properties of graphene make it a suitable material to develop the sensors, transparent and flexible electrodes, electronic circuits, and thermally and electrically conducting reinforced hybrids, which is not possible in the presence of other conventional fillers [37]. Graphene or functionalized graphene is extensively used to improve the various properties of native polymers. Nanohybrids show better mechanical, thermal, electrical, and biological properties than pure polymers do. This can be attributed to the high aspect ratio of the filler, which provides a better platform for interactions with the polymer matrix [38]. Enhancement in gas barrier property was observed for graphene-based nanohybrids owing to its two-dimensional (2D) sheet structure, which restricts the flow of gases [39]. Moreover, nanohybrids have shown more sustained or targeted drug delivery compared to pure polymers [40].  Figure 1 shows some possible applications of unique graphene or its derivatives [41].

This review describes the salient features of graphene and its biomedical applications such as stimuli-responsive drug delivery, tissue engineering, and antibacterial materials in the presence of different polymer matrices. Different techniques such as in situ polymerization, solution casting, and extrusion were used to fabricate graphene-based nanohybrids for desired applications.

## 2. Salient Features of Graphene

**Figure 2:**
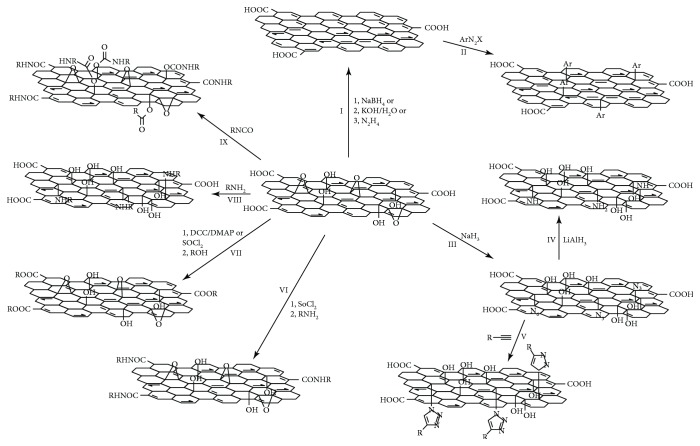
Schematic showing various covalent functionalization chemistries of graphene or GO. I: reduction of GO into graphene by various approaches ((1) NaBH_4_, (2) KOH/H_2_O, and (3) N_2_H_4_). II: covalent surface functionalization of reduced graphene via diazonium reaction (ArN2X). III: functionalization of GO by the reaction between GO and sodium azide. IV: reduction of azide functionalized GO (azide–GO) with LiAlH_4_ resulting in the amino-functionalized GO. V: functionalization of azide–GO through click chemistry (R–ChCH/CuSO_4_). VI: modification of GO with long alkyl chains ((1) SOCl_2_ and (2) RNH_2_) by the acylation reaction between the carboxyl acid groups of GO and alkylamine (after SOCl_2_ activation of the COOH groups). VII: esterification of GO by DCC chemistry or the acylation reaction between the carboxyl acid groups of GO and ROH alkylamine (after SOCl_2_ activation of the COOH groups) ((1) DCC/DMAP or SOCl_2_ and (2) ROH). VIII: nucleophilic ring-opening reaction between the epoxy groups of GO and the amine groups of an amine-terminated organic molecular (RNH_2_). IX: the treatment of GO with organic isocyanates leading to the derivatization of both the edge carboxyl and surface hydroxyl functional groups via formation of amides or carbamate esters (RNCO) [44].

Among various nanomaterials, graphene has a variety of advantages and gained tremendous attention from the scientific community. Graphene is a 2D single atomic layer of graphite with sp^2^-hybridized carbon atoms arranged in a honeycomb structure. It was initially described by Boehm et al. in 1986 followed by identification and isolation by Geim and Novoselov in 2004 [42, 43]. In graphene, each carbon atom is connected by *σ* bonds with a delocalized *π* electron network. These delocalized *π* electrons provide a high electron density above and below the 2D planar structure of graphene. Because of the planar structure and delocalized *π* electrons, graphene undergoes various reactions such as cycloadditions, click reactions, and carbine insertions [44]. Pure graphene is hydrophobic in nature and requires stabilizing agents or surfactants to disperse in water [45]. In addition to graphene, GO and reduced graphene oxide (RGO) are used to improve material properties. Since GO contains several functional groups in its structure, there is a high possibility of surface modification.  Figure 2 shows a few chemical functionalizations of the GO structure [44]. Some salient features of graphene are given in the next sections.

## 3. Mechanical Properties

Several techniques such as force displacement, force volume, nanoindentation atomic force microscopy (AFM) [46–48], and numerical simulation [49–51] are used to determine the mechanical strength of the wonder graphene material. It is observed that defect-free single layer graphene is a much stronger than steel [52]. The Young's modulus, fracture strength, and Poisson's ratio of a defect-free single layer graphene are 1 TPa, 130 GPa, and 0.149 GPa, respectively [52]. On the other hand, GO has several defects and thus shows significantly lower mechanical strength than that of a defect-free graphene with a Young's modulus of 0.15–0.35 TPa [53, 54], elastic modulus of 32 GPa, and fracture strength of 120 MPa [54]. The mechanical strength of defective GO films can be improved by the reduction process or using cross-linking agents [55]. Since graphene has exceptional mechanical strength, it is widely used to enhance the mechanical strength of polymeric materials for various applications. Besides, it was observed that graphene with other nanomaterials such as carbon nanotubes cause greater improvement in the mechanical strength of polymeric materials with individual nanomaterials due to the synergistic effect of both the nanomaterials [56].

## 4. Thermal and Electrical Properties

The presence of strong *σ* bonding and delocalized *π* electrons makes graphene a unique electrically and thermally conductive material with a low thermal expansion coefficient. The thermal conductivity of defect-free graphene is much higher than that of other carbon nanomaterials. Its thermal conductivity is approximately in the order of 4.5–5.5 × 10^3^ W/mK, whereas it is approximately 2 × 10^3^, 3 × 10^3^, and 3.5 × 10^3^ W/mK for graphene oxide, multiwalled carbon nanotubes, and single-walled carbon nanotubes, respectively [52, 57, 58]. The thermal conductivity of GO is lower than that of graphene due to the presence of defects in its structure that hinders the conductivity. This property is greatly influenced by several factors such as doping or defect edge scattering, which cause localization of phonons [59–61]. The electrical mobility of defect-free graphene is higher than that of defective GO and is in the order of 10^4^ S/cm. The electrical mobility of GO is 10^−1^ S/cm [62]. With its excellent inherent thermal and electrical properties, graphene is extensively used in the fabrication of low-cost and highly efficient electronic devices. In addition, it is used in tissue engineering, biosensors, and other biomedical devices to measure the cell potential [63, 64].

## 5. Optical Properties

**Figure 3:**
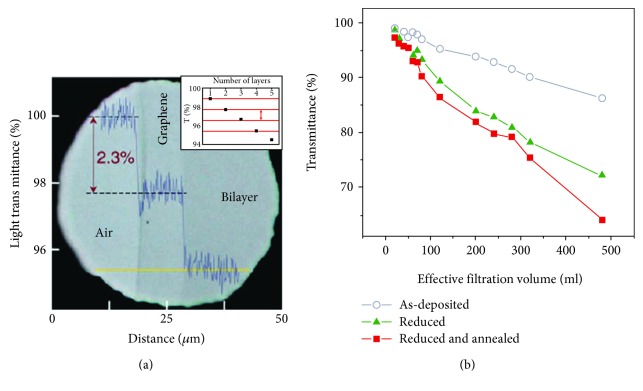
Optical transparency. (a) Optical micrograph of one- and two-atom-thick graphene crystals. The optical transmittance at 550 nm as a function of the lateral coordinate shows clear steps, the height of which is proportional to the hyperfine constant. In the inset, the linear variation of the transparency as function of number of layers is observed up to six layers. (b) Transmittance at *λ*~550 nm as a function of the thickness of reduced GO thin films, assessed indirectly by the total volume of filtered suspension. Plots are shown for thin films with different reduction steps [66].

In addition to exceptional mechanical strength and thermal and electrical properties, graphene has an excellent optical property. Single layer defect-free graphene has shown 97.7% incident light transmission over a wide range of wavelengths [65]. This property is highly affected by the presence of impurities as well as the number of graphene layers.  Figure 3 shows the optical transparency of one- and two-layer graphene sheets [66]. The excellent optical properties, as well as the superior conductivity of graphene-based materials, open a new dimension to replace the expensive ITO films. High optical transparency, superior conductivity, excellent mechanical strength, and chemical stability make graphene suitable for use as transparent electrodes in solar cells or liquid crystals as well as processable flexible transparent electrodes [67–69]. Photocurrent can be generated by applying an external or internal field during light absorption by the graphene surface. It has been observed that nanosized graphene such as quantum dots have an excellent photoluminescence property. The photoluminescence behavior is highly influenced by the electron-hole pair density in graphene. Higher transmittance and photoluminescence behaviors make graphene the most promising and appealing nanomaterial for application in magnetic resonance imaging (MRI) and biomedical imaging [41].

## 6. Biomedical Applications of Graphene-Based Nanohybrids

### 6.1. Stimuli-Responsive Drug Delivery

**Figure 4:**
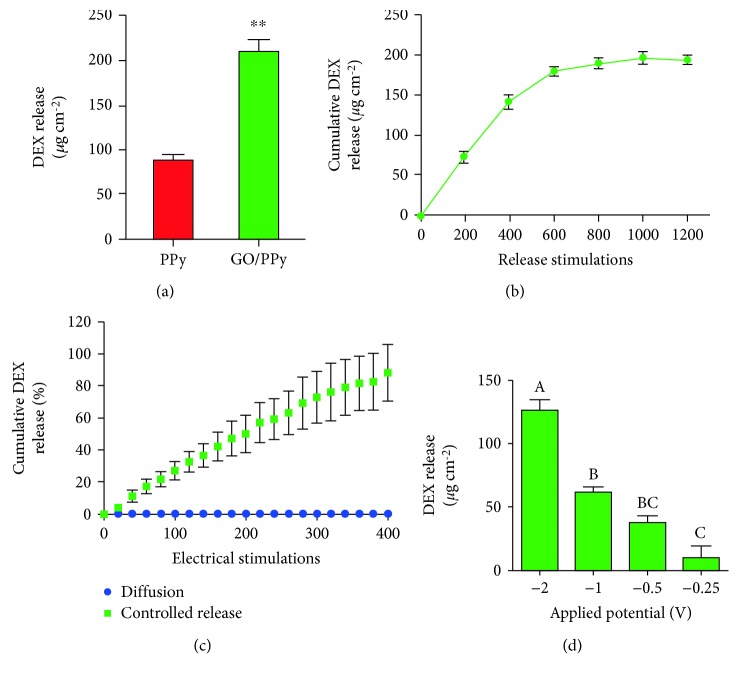
Electrically controlled DEX release from the GO/PPy nanocomposite film. (a) Total DEX release from PPy films with or without GO as a codopant in response to an aggressive square-wave, biphasic voltage stimulation (-2.0 V for 5 s, followed by 0 V for 5 s) repeated for 1000 stimulations. The GO/PPy-DEX nanocomposite released a significantly larger quantity of DEX (*p* < 0.01; *n* = 3). (b) Cumulative release profile of the GO/PPy-DEX nanocomposite in response to aggressive repeated square-wave, biphasic voltage stimulation (-2.0 V for 5 s, followed by 0 V for 5 s) for 1200 stimulations (*n* = 6). The release profile reaches a plateau at 600-voltage pulses under this aggressive stimulation paradigm, indicating that all available drugs have been released at this point. (c) Cumulative release profile of the GO/PPy-DEX nanocomposite in response to milder release stimulation (-0.5 V for 5 s, followed by 0.5 V for 5 s) and in the absence of electrical stimulation (passive diffusion) (*n* = 3). Electrical stimulation elicited a linear release for up to 400 pulses, while no drug passively diffused from the film when no voltage stimulation was applied. (d) Effect of voltage stimulus modulation on the amount of DEX released from nanocomposite films. GO/PPy-DEX nanocomposite films were submitted to 100 square-wave, biphasic stimulation pulses where the negative phase was varied from -2 to -0.25 V, the positive phase was 0.5 V, and the stimulus lingered at each phase for 5 s. Bars labeled with nonmatching letters indicate a significant difference between groups (*p* < 0.01, *n* = 3) [73].

**Figure 5:**
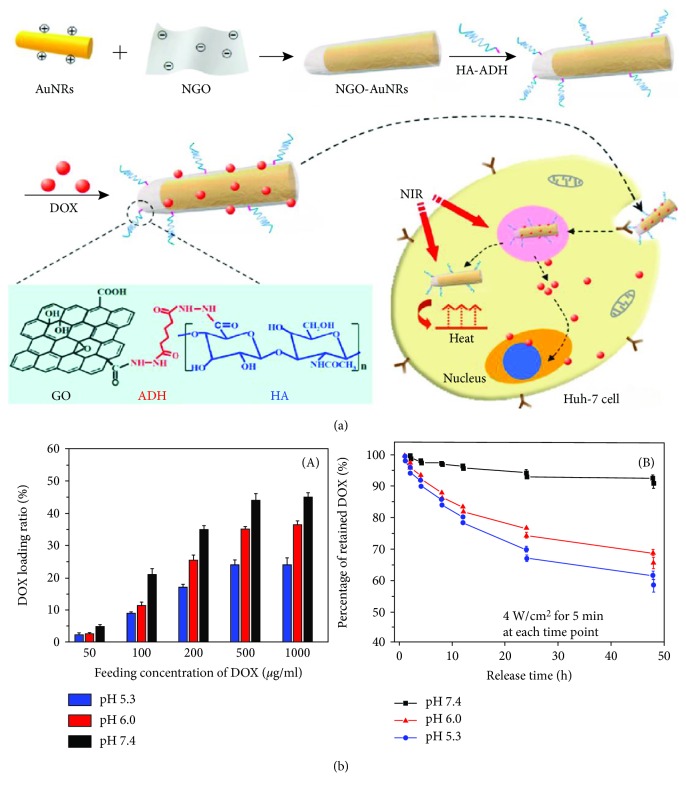
(a) Schematic illustration for the synthesis of NGOHA-AuNRs-DOX and the possible mechanism in targeted chemophotothermal therapy to hepatoma Huh-7 cells. (b, A) pH-dependent DOX loading efficiency of NGOHA-AuNRs at different DOX feeding concentrations. (B) Cumulative release profiles of DOX from NGOHA-AuNRs-DOX at different pH values with 4 W/cm^2^ NIR light irradiation at each time point for 5 min. Data represent mean values for *n* = 3, and the bars are standard deviations for the means [76].

**Scheme 1:**
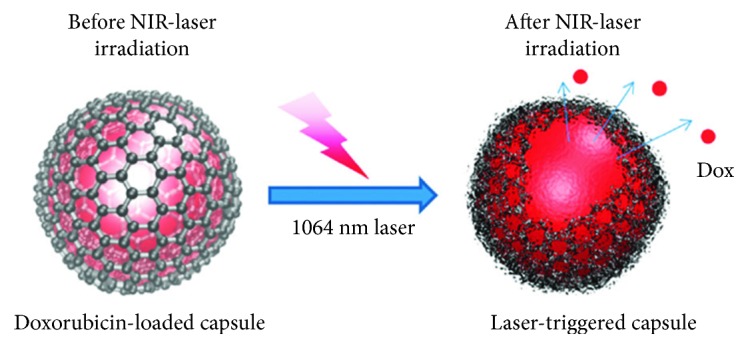
Illustration of the remote opening of GO–polymer composite capsules using NIR-laser light [78].

**Figure 6:**
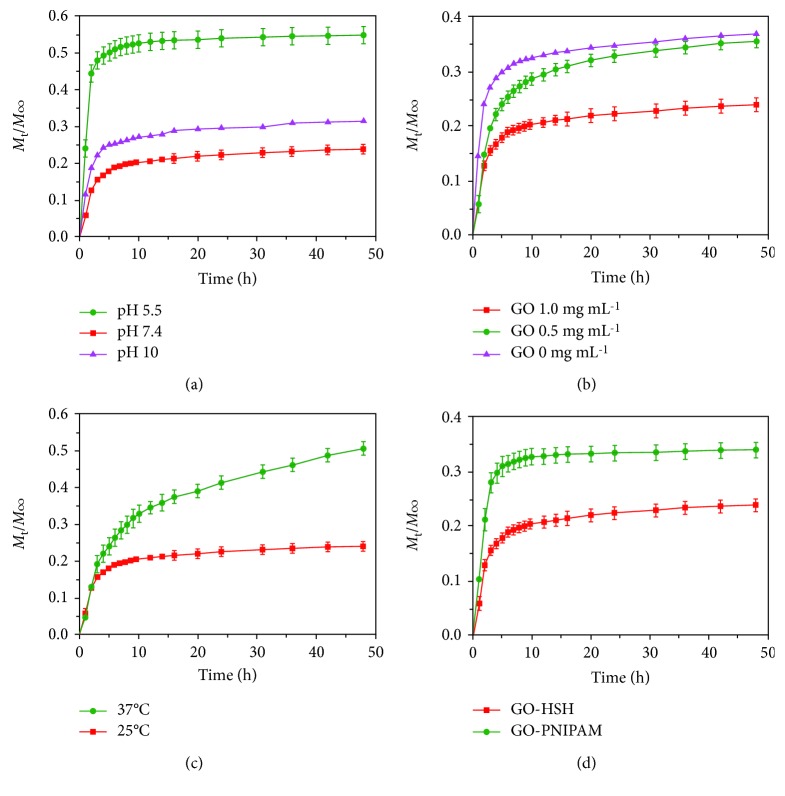
Stimuli-responsive release behavior of DOX from the GO-HSH hydrogel. (a) pH 5.5, pH 7.4, and pH 10 medium at 37°C; (b) varied concentrations of GO nanosheets at 37°C and (c) at 25 and 37°C; (d) DOX release curves of the GO-HSH and GO–PNIPAM hydrogels at 37°C [81].

On-demand or targeted drug release from biomedical devices has attracted great attention in the field of medical science. It has been noted that targeted drug release from carrier molecules exhibits high efficiency with a controllable release and minimum side effects. Several factors such as light, heat, pH of the medium, ultrasound waves, and electric or magnetic fields are responsible for the controlled release of drugs [70–72]. For this purpose, graphene-based nanocarriers are frequently used owing to their large surface areas that facilitate easy loading of drugs and the presence of functional groups provides additional multiple modification routes for targeted and controlled drug release [33, 34]. Nevertheless, care should be taken so that no toxic materials are released from the nanocarriers during stimulation. An electrically responsive drug release material was synthesized by Weaver et al. using conducting pyrrole and GO through electropolymerization on glassy carbon electrodes. They loaded dexamethasone drug in this hybrid and evaluated its release behavior under an external electrical field. A linear drug release was observed from the nanohybrids, which could be changed by varying the magnitude of the external electric field. Interestingly, no passive release of loaded drug occurred from the nanohybrids in the absence of an electric field. The drug-release behavior of the nanohybrids can also be optimized by changing the size and thickness of GO. On the other hand, the released drug maintains its bioactivity without the leaching of additional toxic products during electrical stimulation. Since GO nanoparticles are larger than the loaded drug molecules, only small molecules are released from the nanohybrid film during stimulation, while larger materials are intact within the polymer matrix.  Figure 4 shows the controlled release of dexamethasone drug from a GO/poly pyrrole nanohybrid film [73]. Photothermally induced drug release from nanomaterials has gained significant attention in the treatment of cancer to achieve controllable release with high efficiency and minimum side effects during the treatment [74]. Cancer treatment through chemotherapy has many limitations such as low efficacy, side effects, and drug resistance [75]. Xu et al. synthesized photothermally mediated nanocarriers using nano-GO and gold nanorods with the conjugation of folic acid-modified hyaluronic acid. A schematic representation of the synthesis of nano-GO-based hybrids and the possible mechanism in targeted chemophotothermal therapy are shown in Figure 5(a). The pH-dependent loading of anticancerous doxorubicin hydrochloride drug into the hybrids and its release profiles under different pH media are shown in Figure 5(b). It was observed that the loading capacity is higher in an alkaline medium than in a neutral or acidic medium due to the greater hydrophobic interactions between the nano-GO and the anticancer drug. However, a faster drug release behavior was observed in the acidic condition due to protonation of the loaded drug and, consequently, an increase in the water-soluble tendency. This property is very useful in the treatment of cancer cells because both the extracellular environment of a tumor and the intracellular lysosome and endosomes are acidic in nature, which facilitate greater release of the drug. The release profile was also influenced by light, and it was observed that irradiation with a near-infrared (NIR) laser for 30 min in 24 h caused a 3.5-fold increase in drug release than that in the absence of light irradiation. This can be attributed to dissociation of *π*-*π* stacking interactions between the drug and the polymer matrix [76]. In another study, Song et al. fabricated hyaluronic acid/GO hybrids as nanocarriers for targeted and pH-responsive release of the anticancer doxorubicin drug through *π*-*π* stacking and hydrogen bonding interactions. A faster drug release from the nanohybrids was observed at pH 5.3 than at pH 7.4, which indicated its potential as a targeted and pH-mediated anticancer drug delivery vehicle [77]. Kurapati and Raichur synthesized NIR light-responsive GO/poly(allylamine hydrochloride) (PAH) multilayered capsules for remote-controlled drug delivery. The capsule templates were prepared by dextran sulfate- (DS-) doped calcium carbonate.  Scheme 1 shows the remote opening of GO-based hybrid capsules using NIR-laser light [78]. Further, pH-induced site-specific drug delivery through poly(2-(diethylamino) ethyl methacrylate) (PDEA)/GO hybrids was studied by Kavitha et al. The fabricated films exhibited good solubility and stability in physiological solutions. The anticancer drug camptothecin (CPT) was loaded through *π*-*π* stacking and hydrophobic interactions between the drug and the nanohybrids. However, drug release was observed only in an acidic medium but not in basic and neutral media, which are found in a tumor environment; this suggests the formation of a suitable site-specific drug carrier [79]. Hydrogel scaffolds with 2D and three-dimensional (3D) structures have been extensively used in drug delivery and other tissue engineering applications owing to their unique physiological properties. Li et al. synthesized NIR light-mediated on-demand release and reversible cell capture scaffolds using GO/poly(N-isopropylacrylamide) (pNIPAAm) via an in situ atom-transfer radical polymerization technique. They observed that the release profile was highly influenced by the laser light intensity and the presence of GO [80]. In another study, Chen et al. fabricated a self-healing, pH, and light-induced hydrogel using GO and ureidopyrimidinone and *N*-isopropylacrylamide (pNIPAAm) polymer matrices. They noted that a faster drug (doxorubicin hydrochloride) release from the hydrogel occurred in the acidic medium than in the neutral and alkaline media due to the protonation of polar groups. Furthermore, the developed hydrogels exhibited temperature-mediated drug release, which was more controlled at higher temperatures due to dehydration of the hydrogel leading to a more compact structure that hinders the diffusion of the drug.  Figure 6 shows the pH and temperature-induced drug release from graphene-based hydrogels [81].

### 6.2. Tissue Engineering Applications

**Figure 7:**
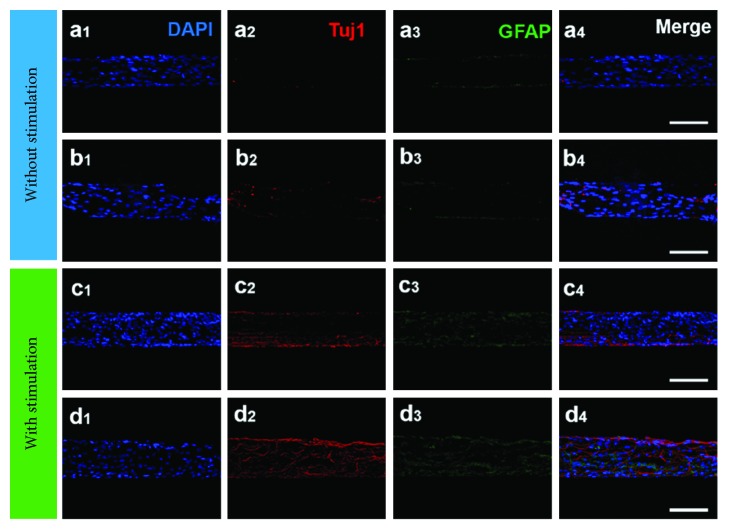
Cells were immunostained with (1) DAPI (blue) for the nucleus and neural-specific antibodies (2) Tuj1 (red, cy3) and (3) GFAP (green, FITC) after being cultured under stimulation culturing conditions without TENG electrical stimulation (a, b) or with human-motion-driven TENG electrical stimulation (c, d) for 21 days on an rGO microfiber (a, c) and 15% rGO-PEDOT hybrid microfiber (b, d). (Right) Merged fluorescence images (scale bar = 100 *μ*m) [89].

**Figure 8:**
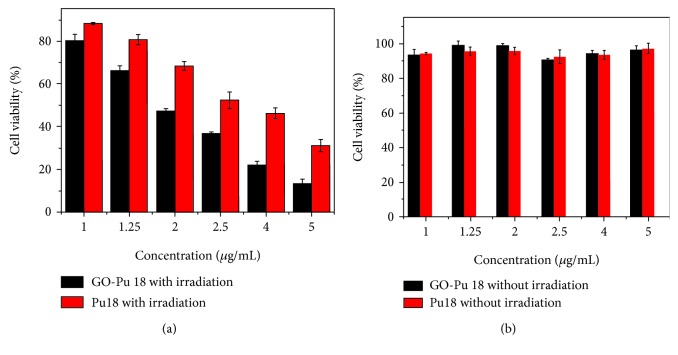
(a) In vitro PDT phototoxicity of GO-Pu18 and free Pu18 to HepG-2 cells and (b) GO-Pu18 composite and free Pu18 to HepG-2 cells without irradiation [93].

**Figure 9:**
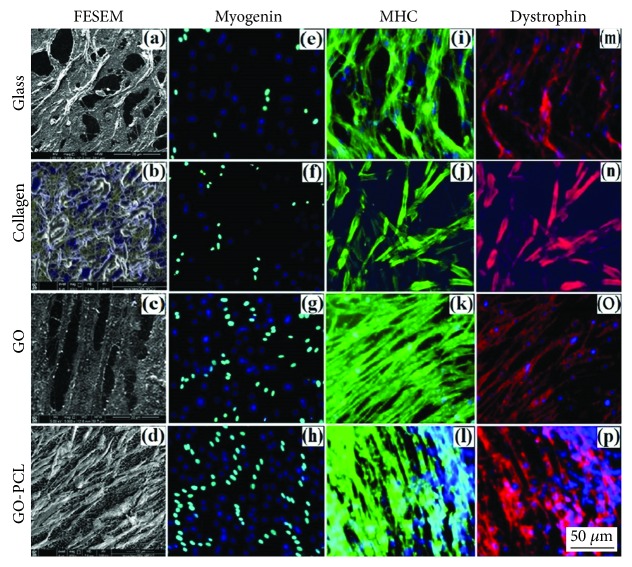
FESEM micrographs showing formation of myotubes on glass and collagen controls (a and b), GO sheets (c), and GO-PCL meshes (d). Expression of the early myogenic differentiation marker myogenin-positive nuclei (green) on controls (e and f), GO sheets (g), and GO-PCL meshes (h). Immunostaining of MHC (green), respectively, on controls (i and j), GO sheets (k), and GO-PCL meshes (l) and dystrophin (red) similarly on controls (m and n), GO sheets (o), and GO-PCL meshes (p). Nuclei were counterstained with DAPI [94].

For tissue engineering applications, materials should be biocompatible, nontoxic, and biodegradable in nature. In addition, materials should not show any negative response in biological conditions [82–84]. Tissue engineering techniques overcome the limitation of traditional medical procedures, wherein repair or replacement of tissues is required. Nowadays, stem cells are most widely studied and used in cell lines for tissue engineering applications owing to their ability to differentiate into various other cells such as osteoblasts and chondrocytes, cardiac muscle cells, neural cells adipocytes, and endotheliocytes in the presence or absence of external stimuli on various surfaces [85–88]. Guo et al. synthesized graphene/poly(3,4-ethylenedioxythiophene) hybrid microfibers and observed its cellular response in the presence of mesenchymal stem cells (MSCs). They noted that neural differentiation of MSCs was dramatically improved by electrical stimulation due to greater interfacial interactions of the electroactive neural cells and the bioelectronic surface, which led to more differentiation of MSCs.  Figure 7 shows the electric-induced cell differentiation of MSCs into neural cells [89]. Similarly, Weaver and Cui demonstrated direct neural stem cell (NSC) differentiation in the presence of a conducting polymer poly(3,4-ethylenedioxythiophene) and GO. They noted that when the surface had interferon-*γ* (INF *γ*) biomolecules, a larger population of neuron cells occurred, while in the presence of a platelet-derived growth factor (PDGF), a larger population of oligodendrocytes occurred, suggesting its potential for controlling the NSC differentiation tendency for therapeutic applications [90]. In another study, Luo et al. fabricated nanofibrous GO/poly(lactic-co-glycolic-acid) hybrids through the electrospinning technique and evaluated its biological responses in the presence of MSCs. A higher cell viability (on the 7th day) and adhesion behavior were observed for the nanohybrid mat compared to the pure polymer due to the strong adsorption of protein onto the nanohybrid surface. In addition, osteogenic differentiation of MSCs occurred on the nanohybrid surface, which was accelerated by GO [91]. Chemical functionalization of graphene plays a crucial role in cellular behavior because it changes the electronic moiety surrounding the graphene sheet that influences the interactions. A comparative study was conducted by Kumar et al. using the GO, RGO, and diamine-modified GO in a poly(*ε*-caprolactone) (PCL) matrix to evaluate the cellular response toward stem cells. They observed that the composite with diamine-modified GO showed a higher proliferation and differentiation of human MSCs (hMSCs) followed by the GO composite. This was due to better interactions between the amine moiety and the cells [92]. Zhang and coworkers also synthesized a pH-sensitive GO conjugate purpurin-18 methyl ester nanocomplex for photodynamic therapy application. A significant decrease in cell viability (HepG-2 cells) was observed in the GO-Pu18 nanohybrids when it was irradiated with light, suggesting that the developed materials have excellent photocytotoxicity and negligible dark response. In vitro photocytotoxicity of the developed material toward HepG-2 cells is shown in Figure 8 [93]. Notably, myoblast differentiation of human cord blood-derived MSCs (CB-hMSCs) into skeletal muscle cells (hSkMCs) were observed on the electrospun fibers of the GO/PCL composite. A high rate of cell proliferation, differentiation, and orientation on the fibrous surface indicated its better biocompatibility. This was due to better interconnections with the fibers and the enhanced conductivity and dielectric properties provided by GO. This property plays a significant role in cell adhesion followed by higher proliferation and myotube orientation. Myoblast differentiation of CB-hMSCs via an early expression of myogenin-positive nuclei is shown in Figure 9 [94]. Further, it was reported that the conjugation of GO with low-molecular-weight polyethylenimine (PEI) enhanced the proliferation and differentiation of hMSCs. Kumar and coworkers synthesized GO/PEI-based composites using poly(acrylic acid) (PAA) as a spacer in a PCL matrix. A significant increase in cell proliferation and differentiation was observed in the composite fibers than in the pure PCL and GO/PEI conjugate. This was attributed to the higher number of amine and oxygen functional groups in the composite that led to better interactions between the cells and the fibrous surface [95]. Sayyar et al. synthesized a conducting graphene/chitosan hydrogel and observed its cellular response. They noted that fibroblast cells on the developed scaffold were healthy, which indicated the biocompatibility of the composite [96]. Hydroxyapatite (HA) is frequently used in bone tissue engineering applications; however, its poor mechanical strength restricts its application in long-term functional materials under load-bearing conditions [97, 98]. The properties of HA can be improved by incorporating reinforcing agents. Liu and coworkers prepared hydroxyapatite/RGO nanocomposites and examined their mechanical and biological activities. An enhanced mechanical behavior with improved proliferation and ALP activity of the human osteoblast cells on the nanohybrid surface suggests its potential for use as a biomaterial [99]. A similar observation was made by Li et al. using nanohydroxyapatite and chitosan-functionalized GO [100]. In another study, enhanced osteogenesis and neurogenesis were observed for hMSCs on chitosan/graphene composite surfaces. This can be attributed to the enhance cell-cell and cell-material interactions that promote the functions of hMSCs [101]. Degradation is also an important parameter that indicates whether the materials are useful or not for specific requirements in biological conditions. Natarajan and coworkers synthesized biodegradable composites using GO and galactitol and studied their biocompatibility. They observed that the developed materials were biocompatible with a stimulated osteogenesis property [102]. In addition, some other promising graphene-based scaffolds applications in the tissue engineering field are represented in Table 1.

## 7. Antibacterial Activities

**Figure 10:**
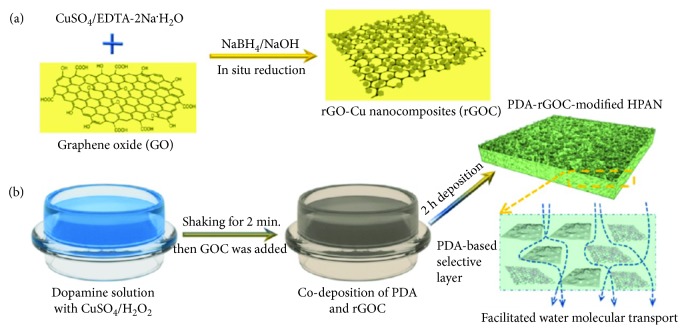
Schematic routes of (a) in situ growth of Cu NPs onto the surface of rGO nanosheets to make rGOC nanocomposites and (b) fast codeposition of PDA and rGOC nanocomposites triggered by CuSO_4_ and H_2_O_2_ [122].

**Figure 11:**
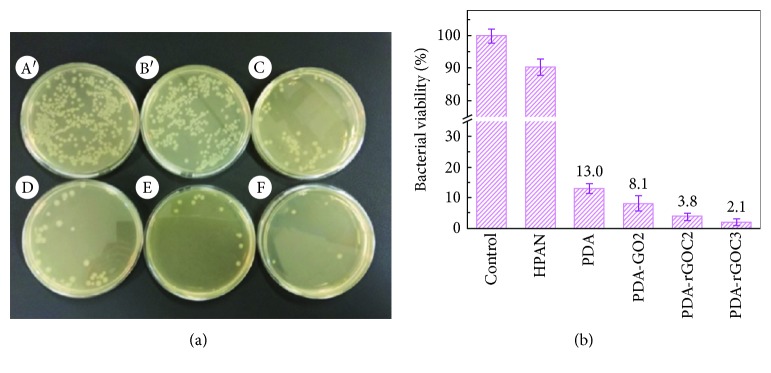
(a) Demonstrated antibacterial properties of the membranes based on the plate counting method: (A′) control without membrane, (B′) HPAN membrane, (C) PDA membrane, (D) PDA-GO2 membrane, (E) PDA-rGOC2 membrane, and (F) PDA-rGOC3 membrane. (b) Quantified antimicrobial ability of the HPAN, PDA-modified, and codeposition-modified membranes [122].

**Figure 12:**
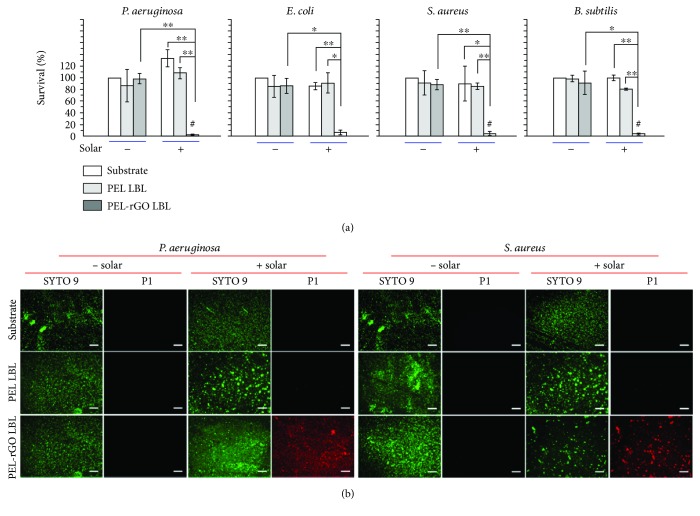
(a) CFU-counting antibacterial assays against four wild-type (wt) bacterial strains consistently reveal that the PEL-rGO LBL thin film, though barely bactericidal when in the dark, killed >90% airborne bacteria on contact within 10 min upon solar irradiation (AM 1.5 G, at one sun). In contrast, the PEL LBL multilayer barely affected bacterial survival no matter whether solar irradiation was applied or not, similar to the behavior of the bare quartz substrate. Data points are reported as mean ± standard deviation. ∗ and ∗∗ indicate *p* < 0.05 and *p* < 0.01, respectively. (b) Bacterial dead/live viability assays under fluorescence microscopy show that *P. aeruginosa* and *S. aureus* cells on a PEL-rGO LBL thin film after 10 min irradiation with a solar simulator (AM 1.5 G) stained intensely red, indicative of dead cells with compromised membranes, whereas those treated similarly but without solar irradiation remained dark in the red channel, indicative of live cells with intact membranes. In striking contrast, cells on a PEL LBL thin film or a quartz substrate remained dark in the red channel in similar assays, no matter whether solar irradiation was applied or not. Scale bar = 100 *μ*m [125].

Nowadays, several antibiotics and antimicrobial agents have been developed for the treatment of various infectious diseases. However, lethal microorganisms remain a challenge for public health, causing several infectious diseases annually [103]. Antibiotics are frequently used to minimize the effect of these pathogens. Moreover, due to the excess use of antibiotics, these pathogens are becoming multidrug resistant [104]. Recently, nanomaterials have gained tremendous attention in this area owing to their unique physical and antibacterial properties that are absent in their macroscopic forms [105]. Various nanomaterials such as graphene, gold, silver, copper, zinc oxide, and magnesium are frequently used for this purpose [106–110]. As mentioned earlier, graphene has drawn wide attention in materials science research owing to its excellent physiochemical and biocompatible properties. Some research works demonstrated that pure GO does not have any antibacterial, bacteriostatic, or cytotoxic properties toward bacteria or mammalian cells [111]. Zhao and coworkers synthesized poly(ethylene glycol)- (PEG-) conjugated GO/silver nanoparticle-loaded composites and evaluated their stability and antibacterial activity. They noted that the composites of PEG-conjugated GO with silver nanoparticles were more stable (over 1 month) than the GO/silver nanoparticle composite was. In addition, they observed that GO-PEG-Ag composites showed more antibacterial activity compared to GO-Ag composites toward Gram-negative/positive bacteria such as *E. coli* and *S. aureus* (~100% of *E. coli* and ~95.3% of *S. aureus*) by 10 *μ*g/mL for 2.5 h. The higher antibacterial activity of GO-PEG-Ag composites was due to the damage of the bacterial structure and the production of reactive oxygen species, which led to cytoplasm leakage and decrease in metabolism [112]. Some et al. synthesized GO-based poly(_L_-lysine) (PLL) composites through electrostatic interactions and covalent bonding between the graphene derivatives and PLL and evaluated their cytotoxicity and antibacterial behavior. They observed that the composites showed a strong antibacterial nature and biocompatibility toward human adipose-derived stem cells and non-small-cell lung carcinoma cells (A549), which indicated its dual functionality that can be used to inhibit bacterial growth as well as enhance human cell growth [113]. In another study, Shao and coworkers synthesized silver nanoparticle-embedded graphene oxide nanocomposites and observed its antibacterial property toward Gram-negative *E. coli* (ATCC 25922) and Gram-positive *S. aureus* (ATCC 6538) by the plate count method and disk diffusion method. Significant antibacterial activity was observed for the nanocomposites, which suggested its potential use in biomedical applications [114]. Microbial contamination such as waterborne pathogens including bacteria, protozoans, helminthes, fungi, and viruses cause several severe diseases to human beings [115]. Several techniques such as ultraviolet (UV) treatment and chemical and thermal treatments are frequently used for water purification processes [116]. Nanofiltration (NF), one of the most studied membrane technologies for a wide range of applications such as water purification/desalination, textile dyes/heavy metals/natural organic removal, and oil/water separation, uses membranes with pore sizes of 0.5–2 nm [117–121]. Zhu et al. prepared a nanofiltration membrane based on RGO and copper nanoparticles through an in situ reduction process on a polydopamine (PDA) surface and evaluated its dye purification or desalination behavior with antibacterial performance.  Figure 10 shows the schematic of the synthesis routes to the nanocomposite and its deposition on a PDA surface. A PDA-rGOC-modified membrane exhibited strong antibacterial property toward *E. coli* (~97.9% reduction) after 3 h of contact, indicating its multidynamics applications with strong antibacterial and separation performances. The antibacterial activity of the PDA-rGOC-modified membrane is shown in Figure 11 [122]. Musico et al. modified the commercially available cellulose nitrate membrane filter papers with poly(*N*-vinylcarbazole) (PVK) and graphene/GO. The PVK-GO-modified membrane exhibited a strong antibacterial activity toward *B. subtilis* and *E. coli.* This was due to the production of reactive oxygen species by the nanoparticles, which influenced the metabolic activity of the microorganisms [123]. Liu et al. studied the antibacterial activity of a polylactic acid-GO-silver nanoparticles hybrid toward *S. aureus* [124]. It is well known that graphene has a high tendency to absorb NIR light and reflect it in the form of heat. This property of graphene has a wide range of applications in materials science. A light- (NIR-) induced antibacterial surface was prepared using PEI and RGO on a quartz surface through the layer-by-layer assembly technique. It was observed that >90% airborne bacteria were killed by the developed surface on exposure to light.  Figure 12 shows the light-induced antibacterial activity of a PEI-rGO thin film synthesized by the layer-by-layer technique [125]. A similar study was carried out by Xie and coworkers in the presence of GO/Ag nanoparticles wrapped with a thin layer of type I collagen under 660 nm visible light irradiation. Approximately 96.3% and 99.4% of *E. coli* and *S. aureus* bacteria, respectively, were killed by the developed hybrids under irradiation of 660 nm light due to the formation of radical oxygen species; this indicated the strong photocatalytic activity of the hybrid toward microorganisms [126]. Konwar et al. fabricated graphene- (GIO-) based hydrogels using chitosan as a polymer matrix via a gel-casting technique and evaluated its antimicrobial activity against *S. aureus*, *E. coli*, and *C. albicans.* A significant improvement in antimicrobial activity was observed for the GIO-based hydrogel film compared to chitosan-GO and chitosan-iron oxide films [127]. Antibacterial and photocatalytic activities were also observed for a three-phase TiO_2_/Ag_3_PO_4_/graphene composite synthesized by an ion-exchange method and a hydrothermal approach [128]. Materials with antimicrobial activity have drawn wide attention in wound healing applications owing to their ability to kill pathogens at a wound site. Dubey and Gopinath fabricated multicomponent composites based on silver nanoparticles, GO, chitosan, and curcumin. They noted that the fabricated nanofibers have good biocompatibility and better antibacterial activity, which indicated their potential for biomedical applications [129]. Further, a considerable enhancement in antibacterial activity toward *E. coli* and *S. aureus* with negligible cytotoxicity was observed for silver-incorporated ZnO-chemically converted graphene nanocomposites synthesized by a low-temperature technique using zinc acetate dehydrate, silver nitrate, and GO [130].

## 8. Conclusions

Nanomaterials with unique intrinsic physiochemical and biological properties, which are absent in their macro forms, have drawn significant attention in materials science for various applications. Several nanomaterials such as a graphene, carbon nanotubes, fullerenes, zeolite, and metals in different forms are frequently used to improve the native properties of materials for desired applications. Nowadays, graphene, an allotrope of carbon with excellent thermal, electrical, optical, mechanical, and biological properties and a higher surface area, is intensively used to enhance material properties. Moreover, properties of graphene can be tuned by surface functionalization with various groups. The higher surface area of graphene and the high charge density on the graphene surface facilitate the loading of several drug molecules, and they consequently act as a nanocarrier with tune rate in the biological medium. In addition, the excellent physical property of the graphene surface facilitates the proliferation and differentiation of cells. Moreover, its light-absorbing behavior plays an essential role in light-triggered drug delivery or cellular response. A significant improvement in antibacterial activity without cytotoxicity was observed for various graphene-based hybrids, suggesting its potential as a biomaterial for various applications. Hence, a discovery of wonder graphene nanomaterial has opened a new area of research to produce lightweight, high-performance hybrid materials for various biomedical applications.

## Figures and Tables

**Table 1:** Graphene-based scaffolds for tissue engineering application.

Graphene-based scaffolds	Tissue engineering applications	Observations	References
Antibody coated Au-nanoparticles on pyrolytic graphite Graphene oxide-based silk fibroin (SF) nanoparticles	Immunosensor for stem and carcinoma cell through Nanog detection Stem cell differentiation	Good sensitivity (0.1-160 pg/mL) in human embryonic stem cell lysates Accelerated early cell adhesion and induced osteogenic differentiation of hMSCs	[102, 131]

Graphene-coated surfaces, e.g., polydimethylsiloxane (PDMS), glass, and Si/SiO_2_ substrates	Stem cell differentiation	Controlled and accelerated differentiation of hMSCs Accelerated adherence of human osteoblasts and mesenchymal stromal cells	[132–134]

Graphene oxide/graphene oxide-coated surfaces	Culture and differentiation of stem cells	Induced pluripotent stem cell culture and differentiation Improved stem cell adhesion and differentiation	[135, 136]

Graphene foam	Stem cell differentiation	Promotion of osteogenic differentiation of hMSCs Promotion of neural stem cell (NSCs) differentiation into astrocytes and neurons Promotion of in vivo mimicking conditions as well as effective cell adhesion, proliferation, and differentiation towards any desired tissue regeneration Increased cell adhesion, proliferation, and differentiation of neural stem cells (NSCs) Promotion of mouse mesenchymal stem cell (MSC) differentiation toward dopaminergic neurons	[137–141]

Activated charcoal	Stem cell differentiation	Promotion of human embryonic stem cell differentiation toward neuronal lineage	[142]

Fluorinated graphene	Stem cell differentiation	Promotion of human stem cells into neuronal lineage	[143]

Graphene microfiber	Stem cells differentiation	Promotion of adhesion, proliferation, and differentiation of neural stem cells (NSCs)	[144]
